# Real-time quantification of laser speckle contrast imaging during intestinal laparoscopic surgery: successful demonstration in a porcine intestinal ischemia model

**DOI:** 10.1007/s00464-024-11076-3

**Published:** 2024-07-17

**Authors:** J. Tim Hoffman, Danique J. I. Heuvelings, Tim van Zutphen, Laurents P. S. Stassen, Schelto Kruijff, E. Christiaan Boerma, Nicole D. Bouvy, Wido T. Heeman, Mahdi Al-Taher

**Affiliations:** 1 Faculty Campus Fryslân, University of Groningen, Wirdumerdijk 34, 8911 CE Leeuwarden, The Netherlands; 2 University Medical Centre Groningen, Optical Molecular Imaging Groningen, Hanzeplein 1, 9713 GZ Groningen, The Netherlands; 3 Department of Surgery, Medical Centre Leeuwarden, Henri Dunantweg 2, 8934 AD Leeuwarden, The Netherlands; 4 LIMIS Development, Henri Dunantweg 2, 8934 AD Leeuwarden, The Netherlands; 5 NUTRIM, Research Institute of Nutrition and Translational Research in Metabolism, Maastricht University, Universiteitssingel 50, 6229 ER Maastricht, The Netherlands; 6 Department of Surgery, Maastricht University Medical Centre+, P. Debyelaan 25, 6229 HX Maastricht, The Netherlands; 7 GROW School for Oncology and Developmental Biology, Maastricht University, Universiteitssingel 50, 6229 ER Maastricht, The Netherlands; 8 Department of Surgery, University Medical Centre Groningen, Hanzeplein 1, 9713 GZ Groningen, The Netherlands; 9 Department of Nuclear Medicine and Molecular Imaging, University Medical Center Groningen, Groningen, The Netherlands; 10 Department of Molecular Medicine and Surgery, Karolinska Institutet, Solnavägen 1, Solna, 171 77 Stockholm, Sweden

**Keywords:** Anastomotic leakage, Image-guided surgery, Laparoscopic surgery, Laser speckle contrast imaging, Perfusion assessment

## Abstract

**Background:**

Anastomotic leakage (AL) is a dreaded complication following colorectal cancer surgery, impacting patient outcome and leads to increasing healthcare consumption as well as economic burden. Bowel perfusion is a significant modifiable factor for anastomotic healing and thus crucial for reducing AL.

**Aims:**

The study aimed to calculate a cut-off value for quantified laser speckle perfusion units (LSPUs) in order to differentiate between ischemic and well-perfused tissue and to assess inter-observer reliability.

**Methods:**

LSCI was performed using a porcine ischemic small bowel loop model with the PerfusiX-Imaging® system. An ischemic area, a well-perfused area, and watershed areas, were selected based on the LSCI colormap. Subsequently, local capillary lactate (LCL) levels were measured. A logarithmic curve estimation tested the correlation between LSPU and LCL levels. A cut-off value for LSPU and lactate was calculated, based on anatomically ischemic and well-perfused tissue. Inter-observer variability analysis was performed with 10 observers.

**Results:**

Directly after ligation of the mesenteric arteries, differences in LSPU values between ischemic and well-perfused tissue were significant (*p* < 0.001) and increased significantly throughout all following measurements. LCL levels were significantly different (*p* < 0.001) at both 60 and 120 min. Logarithmic curve estimation showed an R^2^ value of 0.56 between LSPU and LCL values. A LSPU cut-off value was determined at 69, with a sensitivity of 0.94 and specificity of 0.87. A LCL cut-off value of 3.8 mmol/L was found, with a sensitivity and specificity of 0.97 and 1.0, respectively. There was no difference in assessment between experienced and unexperienced observers. Cohen’s Kappa values were moderate to good (0.52–0.66).

**Conclusion:**

Real-time quantification of LSPUs may be a feasible intraoperative method to assess tissue perfusion and a cut-off value could be determined with high sensitivity and specificity. Inter-observer variability was moderate to good, irrespective of prior experience with the technique.

**Supplementary Information:**

The online version contains supplementary material available at 10.1007/s00464-024-11076-3.

Anastomotic leakage (AL) is one of the most feared complications following colorectal cancer surgery. It negatively impacts surgical outcome, functional results, and quality of life due to reoperation, permanent diversion, or delayed ostomy reversal [1–3]. Besides, AL increases the total clinical and economic burden [4]. Despite advances in pre-operative risk assessment, operative techniques, and postoperative care, the overall incidence of AL has not significantly decreased over the last decades, with an incidence of 1.5 to 23% and mortality rates as high as 29% [1–3, 5, 6].

Several pre-, intra-, and postoperative risk factors for colorectal AL have been described [7]. The consensus is that sufficient perfusion of tissue is a prerequisite to ensure appropriate anastomotic healing [8–10]. An accurate indication of the borderline between the viable and non-viable tissue, i.e., the watershed area, could help surgeons to create optimal anastomosis and mitigate ischemia-related complications [11]. Currently, the majority surgeons determine the location of anastomosis based on vital signs of the bowel (e.g., mucosal color, pulsation in the mesenteric bed, bleeding from resection lines), a subjective strategy that does not take micro-perfusion and collateral circulation into account [11–13]. Therefore, bowel perfusion assessment is a strategy employed to minimize the risk of AL.

At present, most research focuses on bowel perfusion assessment with intraoperative near-infrared fluorescence imaging (NIRF) using indocyanine green (ICG). However, a more recently developed technique is laser speckle contrast imaging (LSCI). Compared to NIRF, LSCI is a dye-free, non-invasive technique which provides real-time blood flow information by detecting the dynamic interference pattern of laser light on moving red blood cells, known as a speckle pattern [12, 14, 15]. Previous studies demonstrated the feasibility of using laparoscopic LSCI to evaluate real-time intraoperative intestinal perfusion [12, 16–18]. However, optimization and fine-tuning of the technology, supported by additional pre-clinical experiments, are necessary to further validate the anticipated clinical usefulness. Although LSCI generates an objective colormap based on quantitative data to visualize perfusion differences, interpretation of the colormap remains subjective. The color on the map does not indicate tissue viability, but flow. Hypothesizing that quantification of data can enhance objectivity and reproducibility, reduce reliance on individual operators, and potentially improve patient outcomes [19–21], the current study was conducted.

The objectives of this study were twofold: firstly, to establish a cut-off value for laser speckle perfusion units (LSPUs) indicative of optimal tissue perfusion and viability, aiming to furnish surgeons with quantitative data to enhance clinical decision-making and secondly, to evaluate inter-observer reliability among both LSCI experts and inexperienced clinicians. Given lactate’s well-established role as a marker for both systemic and local ischemia [22–24], capillary lactate levels were utilized as a reference point in this study.

## Materials and methods

### Animals and surgical procedure

This animal study was performed at the Central Animal Facilities of Maastricht University (Maastricht, The Netherlands). A total of four mature female Landrace pigs were used for this study, in compliance with the regulations of the Dutch legislation concerning animal research, ARRIVE guidelines, and with approval from a local animal ethics committee (DEC-UM; Number: 2017-021-001).

All surgical interventions were conducted with the administration of general anesthesia. An intravenous combination of medications, including 6-mg/kg Zoletil, 0.01-mg/kg/h sufentanyl (Hameln Pharma GmbH, Hameln, Germany), 9-mg/kg/h propofol (B. Braun Melsungen AG, Melsungen, Germany), and 1-mg/kg/h midazolam (Aurobindo, Baarn, The Netherlands), was administered for anesthesia induction. Throughout the procedure, all animals underwent mechanical ventilation to ensure adequate respiration. Ventilation settings were adjusted when necessary to maintain optimal oxygenation and ventilation. Continuous infusion of sufentanyl and propofol was used to sustain anesthesia, with additional doses administered as required during the procedure. At the conclusion of the experiment, euthanasia was performed using a lethal dose of 200-mg/kg Pentobarbital (AST Farma, Oudewater, The Netherlands).

A midline laparotomy was performed by an experienced surgeon and small bowel loops of approximately 20 cm in length were randomly selected. Subsequently, a minimum of eight consecutive mesenterial arteries feeding the loop were identified and ligated using an energy device (Thunderbeat, Olympus, Hamburg, Germany) to induce ischemia.

### Laser speckle contrast imaging

A PerfusiX-Imaging® device (LIMIS Development BV, Leeuwarden, The Netherlands) was used to perform laparoscopic LSCI, as described by Heeman et al. [17]. This is a laparoscopic perfusion imager that functions as an add-on with a range of widely clinically available laparoscopic video equipment. In this study, an OTV-S200 laparoscopic video system (Olympus, Hamburg, Germany) and a 30-degree chip-on-the-tip laparoscope (EndoEye, Olympus, Hamburg, Germany) were used in combination with the investigational device. This setup is capable of instantaneously providing real-time perfusion maps of intestinal tissue using a red laser source. A proprietary mechanism in the device allows switching between the original white light source and the visible red laser light.

LSCI is based on changes in the speckle pattern that arise when illuminated tissue contains moving particles [25]. The level of moving particles (i.e., red blood cells) affects the changes in speckle contrast, allowing for calculation and visualization of perfusion levels through 2D perfusion maps on the surgical monitor. The colormap shows a gradient between blue (relatively low perfusion) and yellow (relatively good perfusion), based on LSPUs (arbitrary units, or AU). LSPU’s were calculated by the ratio of the standard deviation (SD) divided by the mean intensity of the pixels in a window of 7 × 7 pixels. During the procedure, the surgeon was able to view live LSPU values, as well as a graph plotting LSPU values over time. For standardization purposes, the laparoscope was placed in a 3D-printed mount, 14 cm above the specimen, with the camera sensor placed perpendicular to the tissue.

### Data acquisition and statistical analysis

#### LSPUs and lactate levels

**Fig. 1:**
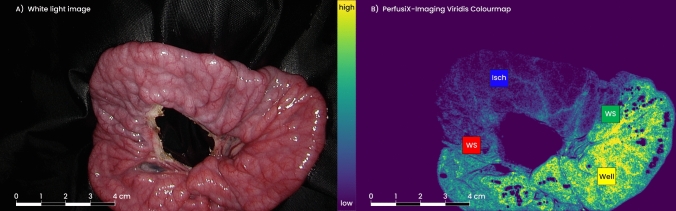
Small bowel tissue was placed in an extracorporeal loop on black drape. At T_0,_ arteries were ligated to induce ischemia. **A** White light image as produced by a standard laparoscopic system. The mesenteric defect in the middle of the loop is the result of cauterization of arterial vascularization. **B** Visualization of perfusion levels in the same intestinal loop as seen by the surgeon during the procedure. Four regions of interest (ROIs) can be seen, representing the surgeon-selected perfusion areas: Well = well-perfused tissue (yellow); WS = watershed areas (red and green); Isch = ischemic tissue (blue). The scale bar on the left of the colormap indicates the low flow (blue) to high flow (yellow) gradient (Color figure online)

Four timepoints were selected to acquire data during the procedure. At T_-1_, prior to any manipulation of vascularization occurred, an LSCI recording of the untouched intestinal loops was made. Each loop was placed outside of the abdominal cavity on a black drape at the time of imaging for standardization purposes. During recording, lights in the operating room (OR) were turned off. Immediately after ligation of the arteries, the LSCI visualization mode was turned on and shown real time to the operating surgeon (T_0_). Following a concise explanation on the system’s visualization of perfusion using a color map, the surgeon designated four regions of interest (ROIs) accordingly: an ischemic area, a well-perfused area, and two watershed areas (transition zones between well- and poorly perfused tissue) (Fig. 1). These ROIs were marked with a surgical tissue marker pen for reference during the image analysis. LSCI recording was repeated 60 (T_60_) and 120 (T_120_) minutes after devascularization. In addition to the LSCI recording, systemic lactate levels were taken at T_0_, T_60_, and T_120_ for every loop to estimate the ischemic state of the pig. Also, local capillary lactate (LCL) levels in the intestinal serosa were measured at the four ROIs. For practical reasons, this was done at T_0_ for the watershed and ischemic ROIs only in 3 loops, but in all loops for the well-perfused ROIs. At T_60_ and T_120_, LCL levels were taken at all four ROIs in all loops. The LCL measurement was done using a 23 Gauge needle and an EDGE lactate analyzer (ApexBio, Taipei, Taiwan, People’s Republic of China) which allowed for instant lactate measurements.

#### Data quality assessment

Sequences were inspected for artifacts, such as erroneous ROI tracking or surgical instruments blocking a clear view on the intestinal tissue. Artifacts were excluded from further analysis. The middle 96 frames of each recording were used to equalize sequence length and prevent any selection bias. ROIs were placed, based on surgical pen markings, and measured 60 × 60 pixels.

#### Cut-off values

In addition to real-time quantitative perfusion values, a cut-off value for LSPUs was calculated, aiding in the identification of well-perfused and ischemic tissue. To estimate this cut-off value, the Youden index was used, a measure for evaluating the effectiveness of diagnostic tests based on sensitivity and specificity. The index ranges from 0 to 1, with a value of 1 indicating a perfect test with no false positives or false negatives and a value of 0 indicating a test that performs no better than chance [26]. First, a cut-off value with the highest Youden Index for LSPU was calculated, based on anatomically ischemic and well-perfused tissue. Tissues with LSPU levels below the cut-off value were classified as ischemic. The same process was repeated to calculate the cut-off value for lactate levels, which was compared to existing evidence to validate the LSPU cut-off calculation.

#### Inter-observer reliability

In this study, the selection of ROIs was done subjectively by the surgeon based on real-time interpretation of the colormap on the monitor. To assess the robustness and reliability of colormap interpretation, an inter-observer variability analysis was performed after the experiment using the LSCI images taken during the surgery. Five LSCI experts and five physicians (surgical residents) with no experience in assessing LSCI images were asked to locate the watershed areas on the LSCI colormaps, as well as the ischemic and well-perfused areas. The inexperienced physicians received an introductory training consisting of two slides on how LSCI works and how to interpret the colormaps, along with one training image and accompanying text on how to place ROIs. Inter-observer distance was registered in centimeters between all assessors, measuring over a midline on the small intestinal loop. A more detailed explanation can be found in supplemental 1. An expert was defined as someone having multiple years of experience in working with or developing LSCI systems.

#### Statistical analysis

Microsoft Excel (Microsoft Excel version 2312, Microsoft Corporation, Redmond, Washington, United States) and IBM SPSS statistics software package (IBM SPSS statistics version 27, IBM Corp, Armonk, New York, United States) were used to perform statistical analyses. A linear mixed effect model was built using a random intercept model with time and ROI location as fixed effects and an interaction term between time and ROI. A scaled identity covariance structure was used, and it was considered adequate to use Restricted Maximum Likelihood (REML) as estimation method, to prevent biasing by the used method. Logarithmic curve estimation was performed to test the correlation between LSPUs and LCL levels and plot a coefficient of determination, or R^2^. Inter-rate reliability was analyzed using Cohen’s Kappa [27]. Differences were considered significant when *p* < 0.05. Figures were produced with PRISM (PRISM version 10.1.0 (316), GraphPad Software LCC, Boston, Massachusetts, United States). Mann–Whitney *U* tests were conducted for non-normally distributed variables, while *T* tests were employed for normally distributed variables. Numerical variables are presented as mean ± SD or median [IQR 25%–75%] where appropriate.

## Results

**Fig. 2:**
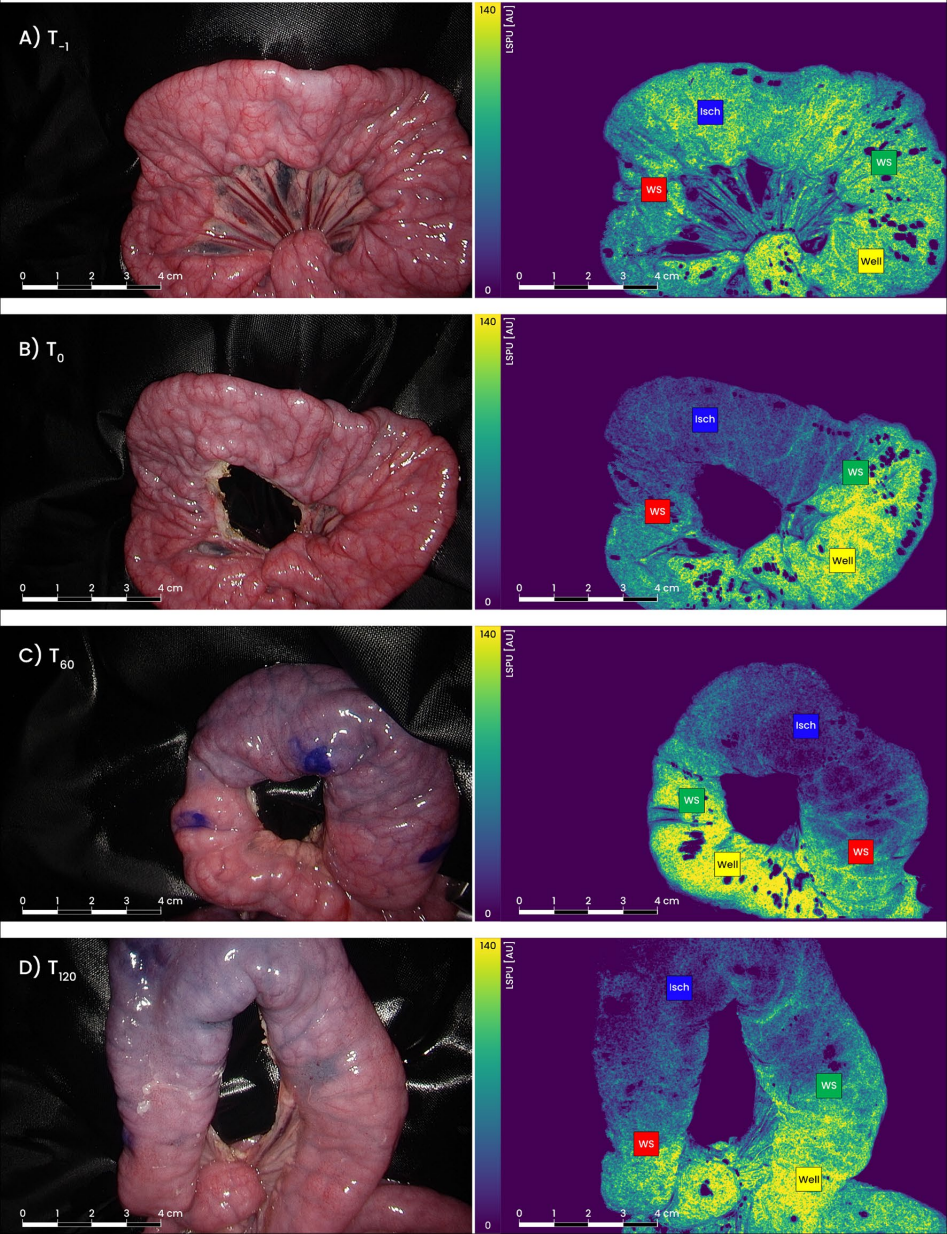
Progression of ischemia over time in both white light images (left) and Laser Speckle Contrast images (right). The Viridis color scheme was used in the visualization of Laser Speckle Perfusion Units (LSPUs). The scale bar on the left of the colormap indicates the low flow (blue) to high flow (yellow) gradient. **A** T_-1_ images show the intestinal loop prior to any vascular manipulation (baseline). ROIs are placed, based on the selection location at the T_0_ measurement (image 2B), since no ischemic and watershed areas could yet be identified. **B** Recording immediately after ligation (T_0_) and selection of Regions of Interest (ROI): Well = well-perfused tissue (yellow); WS = watershed areas (red and green); Isch = ischemic tissue (blue). Locations were marked with a surgical marker for reference. **C** White light and LSCI recording taken 60 min after ischemic onset (T_60_). The blue dots in the white light image are the ROI markings from T_0_. **D** 120 min after ischemic onset (T_120_) (Color figure online)

The surgical procedures were performed without complications or adverse events. The average weight of the landrace pigs was 39 kg (range 36–42). A total of 18 intestinal loops were created, ranging between three and five loops per animal. The operating surgeon was able to interpret all LSCI derived perfusion colormaps in real-time on the surgical monitor and to place the ROIs (Fig. 2).

### LSPU values

**Fig. 3:**
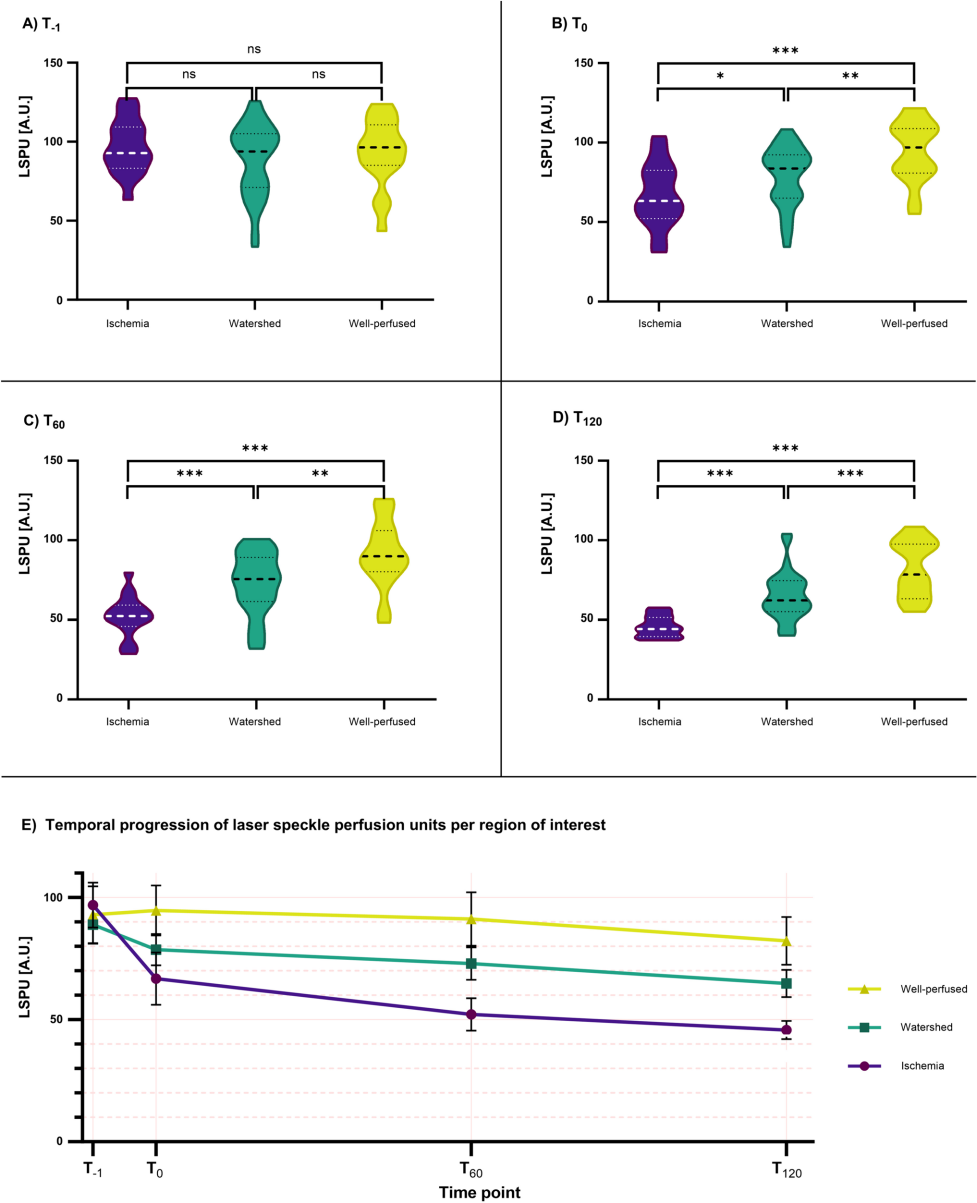
Overview of both spatial (Fig. 3**A**–**D**) and temporal (Fig. 3**E**) progression of average laser speckle perfusion units (LSPUs) per Region of Interest (ROI). **A** LSPUs per ROI before devascularization (T-1). There was no significant difference between the ROIs. **B** LSPUs measured at the ROIs directly after devascularization (T_0_). **C** LSPU measurements after 60 min of ischemia (T_60_). Values further diverge and become more significant. **D** At 120 min after inducing ischemia to the intestinal segment (T_120_), differences between all ROIs are highly significant. Dashed and dotted lines indicate mean values and quartiles respectively. **E** Temporal development of LSPUs per ROI. A strong decline can be seen in ischemic tissue, shortly after inducing ischemia, further decreasing after this. As time progresses, the ischemic ROIs show increasingly low values compared to other ROIs. In addition, 95% CI bars narrow when ischemic time increases. Levels of significance in all images (*p* values): *: ≤ 0.05; **: ≤ 0.01; and ***: ≤ 0.001. An overview of all *p* values can be found in Supplemental 2

At T_-1_, there were no significant differences in mean LSPUs between the watershed, ischemic, and well-perfused areas_,_ as presented in Fig. 3A. At T_0_ (Fig. 3B), and LSPUs of the ROIs started to diverge. Mean ischemic LSPUs were not only significantly lower, compared to well-perfused areas (66.8 ± 19.4 versus 94.7 ± 18.7 AU, *p* ≤ 0.001) but also compared to the watershed areas (78.7 ± 18.3 AU, *p* = 0.038). This difference further increased at T_60_ and T_120_ in all measurements (*p* ≤ 0.004, Fig. 3C–D).

Temporally, all ROIs showed a decrease in perfusion levels (Fig. 3E). Mean LSPUs decreased significantly over time from, respectively, 96.9 ± 8.0 AU at T_−1_ to 45.8 ± 6.4 AU at T_120_ in ischemic areas (*p* ≤ 0.001). In watershed, a decrease from 88.9 ± 7.6 to 64.8 ± 14.8 AU was seen (*p* ≤ 0.001). However, there was no significant change in well-perfused areas over a two-hour period. In ischemic areas, there was a significant decrease of LSPUs in the first hour (66.8 ± 19.4 at T_0_ versus 52.2 ± 12.6 at T_60_, *p* = 0.014). Between T_60_ and T_120_, the curve flattened out. Using a mixed model analysis, the interaction term between time and ROI was significant (*p* ≤ 0.001).

### Lactate levels

Systemic lactate levels ranged from 12 to 30 mmol/L, confirming that none of the pigs experienced systemic ischemia during the experiment. Data collection of the LCL levels missed in one loop at T_120_, resulting in a total of 167 LCL values collected for analysis.

**Fig. 4:**
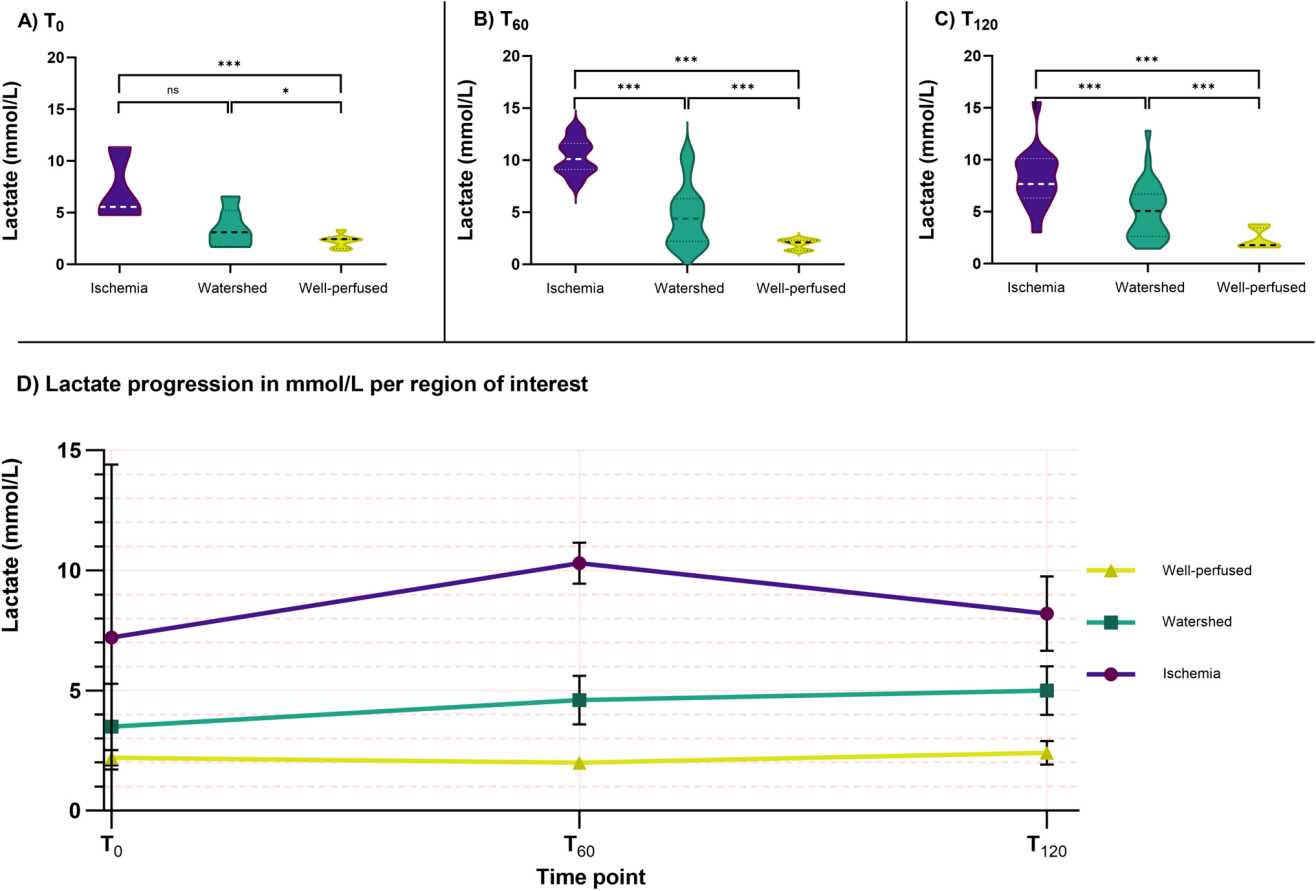
Overview of spatial and temporal differences in lactate (mmol/L) per region of interest (ROI). **A**–**C** Violin plots representing lactate levels per Region of Interest (ROI) per timepoint. No measurements were taken at T_-1_ since ROIs were not yet identified at this moment. Dashed and dotted lines indicate mean values and quartiles, respectively. **D** Temporal representation of mean lactate progression with standard deviation. In the first hour after inducing ischemia, the level of lactate rises significantly in the ischemic area. However, a decrease is seen in the second hour. Well-perfused lactate does not change significantly. Levels of significance in all images (*P* values): *: ≤ 0.05; **: ≤ 0.01; and ***: ≤ 0.001. An overview of all *p* values can be found in Supplemental 2

At T_0_, after ligation of mesenteric arteries, a significant difference in mean LCL levels between well-perfused and ischemic tissue (2.2 ± 0.6 mmol/L and 7.2 ± 1.9 mmol/L, *p* ≤ 0.001) was measured. This difference remained statistically significant over time, with *p* ≤ 0.001 at all timepoints, as can be seen in Fig. 4A–D).

Between T_60_ and T_120_, a significant decrease in LCL levels was seen (10.3 ± 1.6 and 8.2 ± 2.8 mmol/L, respectively, *p* = 0.015). Changes in mean well-perfused tissue lactate were not significant between T_60_ and T_120_ (2.0 ± 0.4 mmol/L versus 2.4 ± 0.9 mmol/L, *p* = 0.1*95)*. Watershed area mean lactate levels showed an increase over time and were significantly higher than well-perfused LCL levels at all given timepoints (Fig. 4). However, these values were significantly lower compared to those in ischemic areas at T_60_ and T_120_ (respectively, 4.6 ± 2.8 versus 10.3 ± 1.6 and 5.0 ± 2.7 versus 8.2 ± 2.8 mmol/L, both *p* ≤ 0.001).

### Cut-off values

**Fig. 5:**
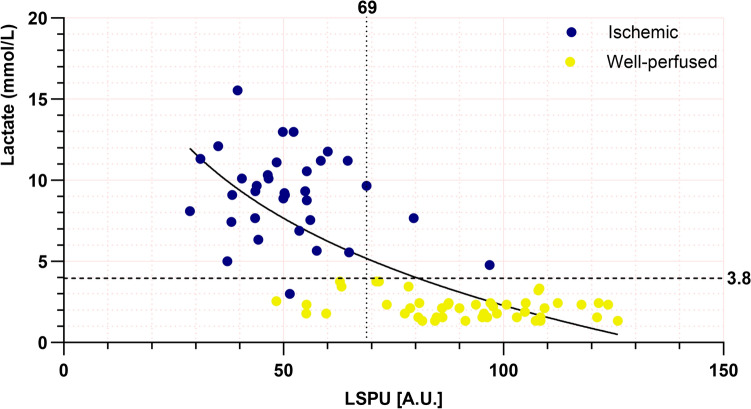
Scatterplot of Regions of Interest (ROI) from ischemic (blue) and well-perfused (yellow) areas. Higher values indicate better perfusion. The horizontal black-dashed line represents the cut-off value for lactate levels (3.8 mmol/L), whereas the vertical dotted line represents the cut-off value for LSPUs (69 AU). In addition, logarithmic curve estimation was used to estimate an R^2^ value of 0.56 (solid black curve). A difference can be noticed between ischemic values and well-perfused values, as higher LSPUs are linked with lower lactate levels (Color figure online)

Logarithmic curve estimation between LSPUs and LCL showed an R^2^ of 0.56. A scatterplot of all ROIs from ischemic and well-perfused tissue with the coefficient can be found in Fig. 5. The cut-off value for LSPUs was determined at 69 AU with a sensitivity of 0.94 and specificity of 0.87 (Youden index 0.81). Consecutively, a cut-off value of 3.8 mmol/L was calculated for lactate, with a sensitivity of 0.97 and specificity of 1.00 (Youden index 0.97).

### Inter-observer variability

**Fig. 6:**
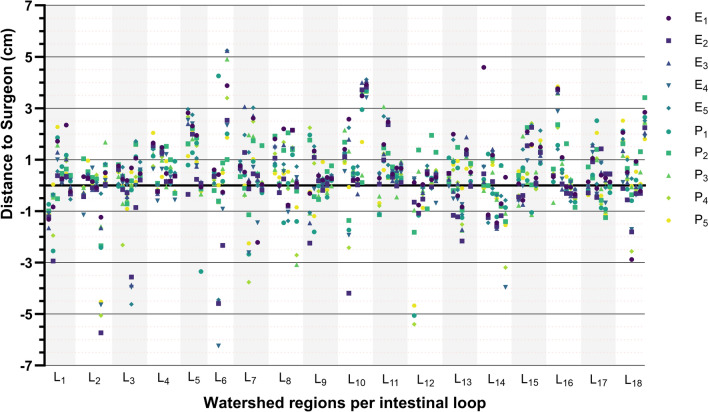
Intra Class Correlation (ICC) dot plot of all assessors per watershed region in all intestinal loops. Distances are measured from the Region of Interest (ROI) placed by the operating surgeon to the ROI placed by a different observer. Negative values are measured from the operating surgeon toward well-perfused tissue. Positive numbers are measured from the operating surgeon toward ischemic tissue. Within each loop, T_0_, T_60_, and T_120_ are shown from left to right, with two ROIs per timepoint (left and right watershed). A more detailed example of how ROI distances are calculated can be found in Supplemental 1. *Ex* expert observer; *Px* Physician, *Lx* loop number

All images were assessed by all experienced and non-experienced observers. No significant differences were observed between the groups regarding the time taken to point out a ROI. Experts failed to place one ROI in 17 cases (3.3%) and physicians in 12 instances (2.3%), citing indistinct transitions between well-perfused and ischemic tissue or broad gradients from high (yellow) to low (blue) values. Both experts and physicians showed similar decision making. A dot plot of all measurements is presented in Fig. 6*.*

When tasked with identifying watershed areas, 66.3% of both experts and physicians placed the ROI more toward the ischemic side (average of 0.54 cm [IQR 0.26–1.41] and 0.66 cm [IQR 0.33–1.29], respectively), compared to the operating surgeon. A site toward better perfused tissue was chosen by 33.5% of experts and 33.7% of physicians (average of 0.44 cm [IQR 1.18–0.18] and 0.45 cm [IQR 1.11–0.18], respectively).

When comparing the placement of all ROIs to that of the operating surgeon, 72% of ROIs were located within one centimeter, whether proximal or distal. In observations within a two centimeter range, this percentage increased to 89%. An inter-rater reliability analysis was conducted comparing the group of experts with the operating surgeon, yielding a substantial Kappa of 0.66 (95% CI 0.58–0.74). The comparison between physicians and the surgeon showed a moderate Kappa of 0.56 (95% CI 0.47–0.65). The comparison between the whole observer group and the surgeon showed a moderate Kappa of 0.52 (95% CI 0.44–0.61, *p* = 0.764).

## Discussion

To our knowledge, this is the first study to correlate quantitative LSPUs to lactate levels and providing a cut-off value for well-perfused tissue, thus adding to the increasing evidence that LSCI may serve as a suitable tool to guide the surgeon in the construction of an optimal anastomosis during laparoscopic surgery [13, 28–32]. Inter-observer agreement among physicians and experts was moderate to substantial, indicating that interpreting LSCI images is feasible even without extensive experience.

A distinct contrast in LSPUs between ROIs was seen at all timepoints following the creation of an ischemic segment. This underscores PerfusiX-Imaging®’s efficacy in visualizing perfusion differences, consistent with previous studies [12, 16–18]. Although there was a clear distinction between areas, all LSPUs continued to decrease over time. In ischemic tissue, this decline is due to diminished flow post-ligation of feeding arteries, restricting blood inflow. Conversely, in well-perfused tissue, intestinal perfusion restriction may result from systemic illness response following prolonged ischemia. In contrast to the LSPUs in ischemic tissue, we surmised that lactate levels decreased after the first hour. This may be attributed to small overlapping vessels on the serosa, originating from a more adequately perfused intestinal segment, facilitating a modest collateral reperfusion effect [33]. This phenomenon may also explain a marginal surge in systemic lactate levels, as collateral vessels transport lactate into the systemic circulation. Additionally, oxygen deprivation from devascularization induces anaerobic fuel consumption, initiating fermentative glycolysis and an initial rise in lactate within ischemic tissue [34]. Compromised metabolic flux in ischemic tissue may deplete glucose supplies, reducing cellular energy consumption and halting lactate formation. Moreover, despite commonly perceived as a waste product, lactate can serve as an alternative fuel source within the tricarboxylic acid (TCA) cycle [35].

Based on our current findings, LSPUs above 69 AU indicate well-perfused tissue, with high sensitivity and specificity, highlighting the robustness of LSCI for perfusion visualization. Nevertheless, extensive additional research is required to extrapolate the use of a cut-off in LSCI in different tissues and humans. The cut-off value for lactate was primarily determined to validate the perfusion areas derived from the PerfusiX-Imaging® device. Values exceeding 3.8 mmol/L appeared indicative of ischemia. Although a well-defined cut-off value for Landrace pigs exists, our results aligned with existing literature, with systemic lactate levels typically below 2.0 mmol/L in a neutral state, while levels exceeding the determined cut-off value were observed in ischemic tissue [12, 33, 36–39].

All evaluators observed clear differentiation between adequately and inadequately perfused tissue. Agreement in identifying watershed regions ranged from moderate to substantial, with no significant difference between experts and unexperienced physicians. However, observers did encounter challenges in interpreting images lacking a clear-cut watershed line. This scenario occurred in case of a more gradual transition between well-perfused and poorly perfused tissue. Consequently, images displaying such transitions exhibited decreased inter-observer agreement, stipulating the importance of quantitative assessment methods to aid in the identification of viable and non-viable tissue.

In contrast to fluorescence angiography, LSCI does not require any pharmaceuticals or dyes, reducing risks of adverse reactions as seen with ICG [40]. Moreover, absence of these substances eliminates the need for timing and dosing calculations and facilitates repeated measurements without the interference of wash-out effects or residual signals [41, 42]. Quantifying ICG poses challenges, as it demands the standardization of numerous factors to obtain meaningful quantitative data [20]. While maximizing standardization in measurements is essential across all modalities, LSCI could emerge as a more direct, real-time, and repeatable approach in providing quantitative information on tissue perfusion [28, 40, 42–45].

### Strengths and limitations

This study maintained standardized conditions [20, 46]. The laparoscope was set at a perpendicular position at 14 cm to tissue to mitigate the effects of camera angulation and laser intensity [13]. PerfusiX-Imaging® utilized algorithms to compensate for motion-induced pixel contrast variations [47]. However, caution is advised in interpreting the findings, and further human investigation is required to assess reproducibility in clinical settings. The device is currently limited to research applications and the intraprocedural LSPU graph presentation is developed specifically for this study. While most of the elective abdominal clinical procedures involve laparoscopic approaches, the bowel loops were created during laparotomy and the laparoscopic system was used in an open setting to maximize standardization (and minimize the procedural time). Nonetheless, a prior technical demonstration has affirmed that the camera system was working appropriately in a total laparoscopic setting [18]. This study specifically addresses ischemia of the small intestine instead of colon surgery, which is more prevalent in daily clinical practice, acknowledging the impracticality of generating ischemic bowel loops in the porcine colon due to its spiral-like orientation [12]. Additionally, colorectal resection in a human population is often complicated by a higher presence of visceral fat compared to a porcine model. Nevertheless, prior studies have demonstrated favorable outcomes utilizing the same device for assessing colonic perfusion in human subjects [16].

Despite the modest sample size, we considered it adequate to address our hypothesis while adhering to the principles of the 3R framework (replace, reduce, refine) in animal research [48]. However, this precluded examination of inter-animal differences from the mixed model analysis. A larger-scale study could offer insights into variations in baseline perfusion and enhance understanding of cut-off values and LSCI quantification.

The next phase would be to further quantify measurements to precisely identify (non-)viable tissue and safe resection zones. Interpreting subtle perfusion differences is crucial for assessing tissue viability, particularly when ischemia is not evidently clear or when achieving this necessitates a profound comprehension of perfusion variations within tissues and across patients, emphasizing the importance of continued studies on the quantification of LSCI. Furthermore, future research should focus on evaluating LSCI in clinical trials to assess its impact on surgical outcomes, including AL rates, and compare its effectiveness with conventional white light imaging.

## Conclusion

This study demonstrates the accuracy of Laser Speckle Contrast Imaging in visualizing and distinguishing between ischemic and well-perfused tissue. Changes in Laser Speckle Perfusion Units corresponded with alterations in lactate levels in both types of tissue. A cut-off value of 69 for LSPU showed high sensitivity and specificity. LSCI holds promise as an adequate and objective perfusion visualization tool, but further research on real-time quantification of LSPUs and clinical applicability is imperative.

## Supplementary Information

Below is the link to the electronic supplementary material.

Supplementary file1 (DOCX 2847 KB)

## Data Availability

Data supporting this study are included within the manuscript and supporting materials. More detailed information can be gained through contacting the corresponding author.
