# Supplementary material for: Real-time quantification of laser speckle contrast imaging during intestinal laparoscopic surgery: successful demonstration in a porcine intestinal ischemia model
Source: Surg Endosc. 2024 Jul 17;38(9):5292–303. doi: 10.1007/s00464-024-11076-3 (PMC11362390; doi:10.1007/s00464-024-11076-3)
Supplement: Supplementary file 1 — Supplementary file1 (DOCX 2847 KB) [file 464_2024_11076_MOESM1_ESM.docx]

**Supplementals**

## Inter observer measurements and calculations.

An Inter-observer reliability analysis was performed with five LSCI experts and five clinicians with no prior experience in interpreting LSCI images (Figure S1). ROIs were placed in well perfused tissue, ischemic tissue and in the transition zones. A line was then plotted over the intestine, indicating equal distance to any pair of sides of the small bowel loop. Using perpendicular lines, the distances from the surgeon’s ROI to the ROI of the observers were measured for the watershed areas (an example can be found in figure S2). Distances where the observer’s ROI was placed towards ischemic tissue were noted as positive values, whereas an ROI towards well-perfused tissue was noted as negative value. This resulted in a violin plot of each observer compared to the surgeon (Figure S3).

In addition, a distribution graph was plotted, indicating the distribution of distances between observers and the surgeon (Figure S4).


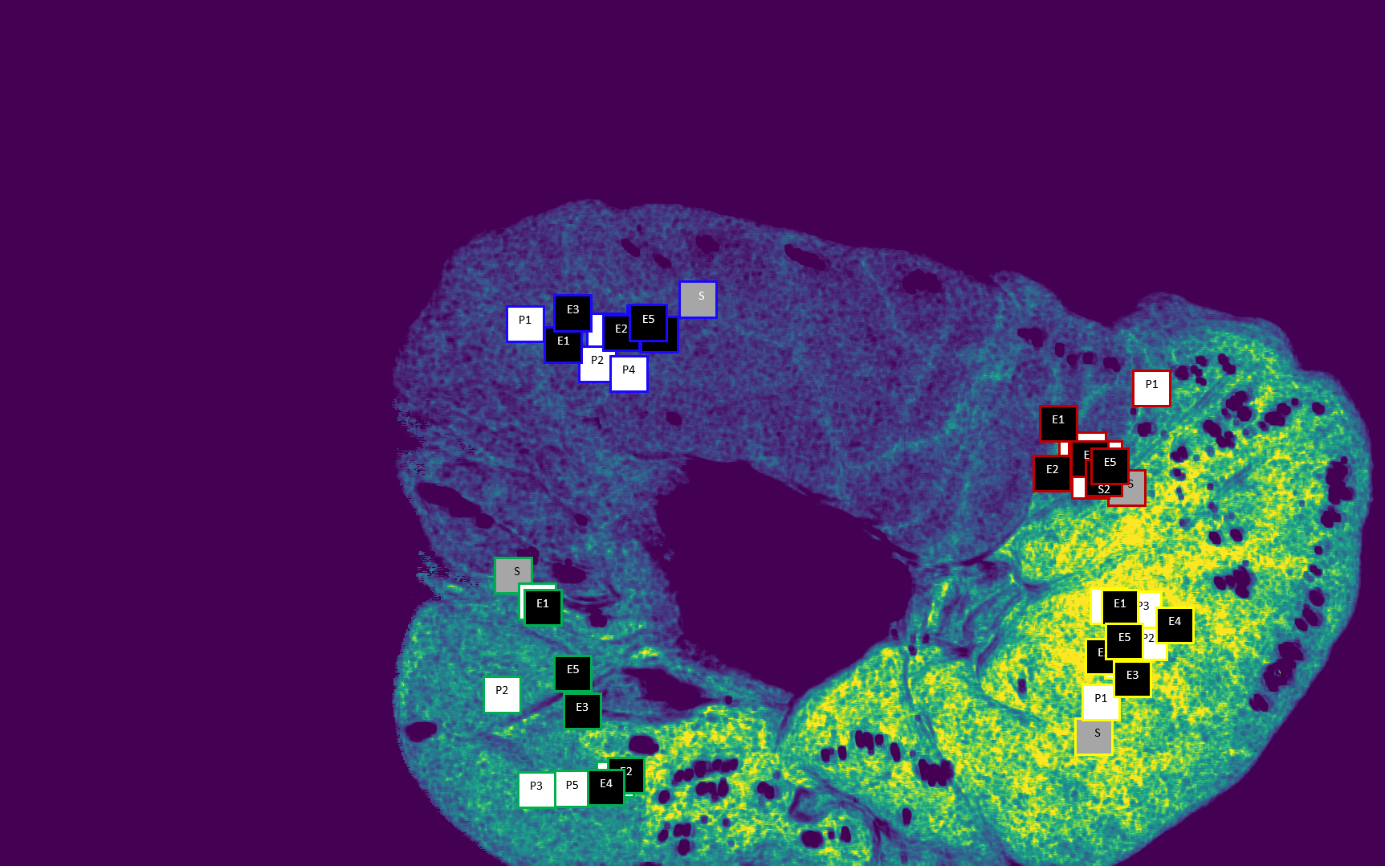


**Figure S1** | Example of an LSCI image, evaluated by all observers. Each square denotes a Region of Interest (ROI) marked by the respective assessor. ROIs outlined in white were placed by physicians, those in black by experts, and the grey ones by the operating surgeon. The blue outline indicates ischemic ROIs, yellow outlines represent well-perfused ROIs, and green and red delineate watershed areas. Notably, the red watershed area on the right exhibits a visually high level of agreement among assessors, while the green watershed area on the left demonstrates comparatively lower agreement. This discrepancy may be attributed to a less distinct transition between well-perfused and ischemic regions in the latter.


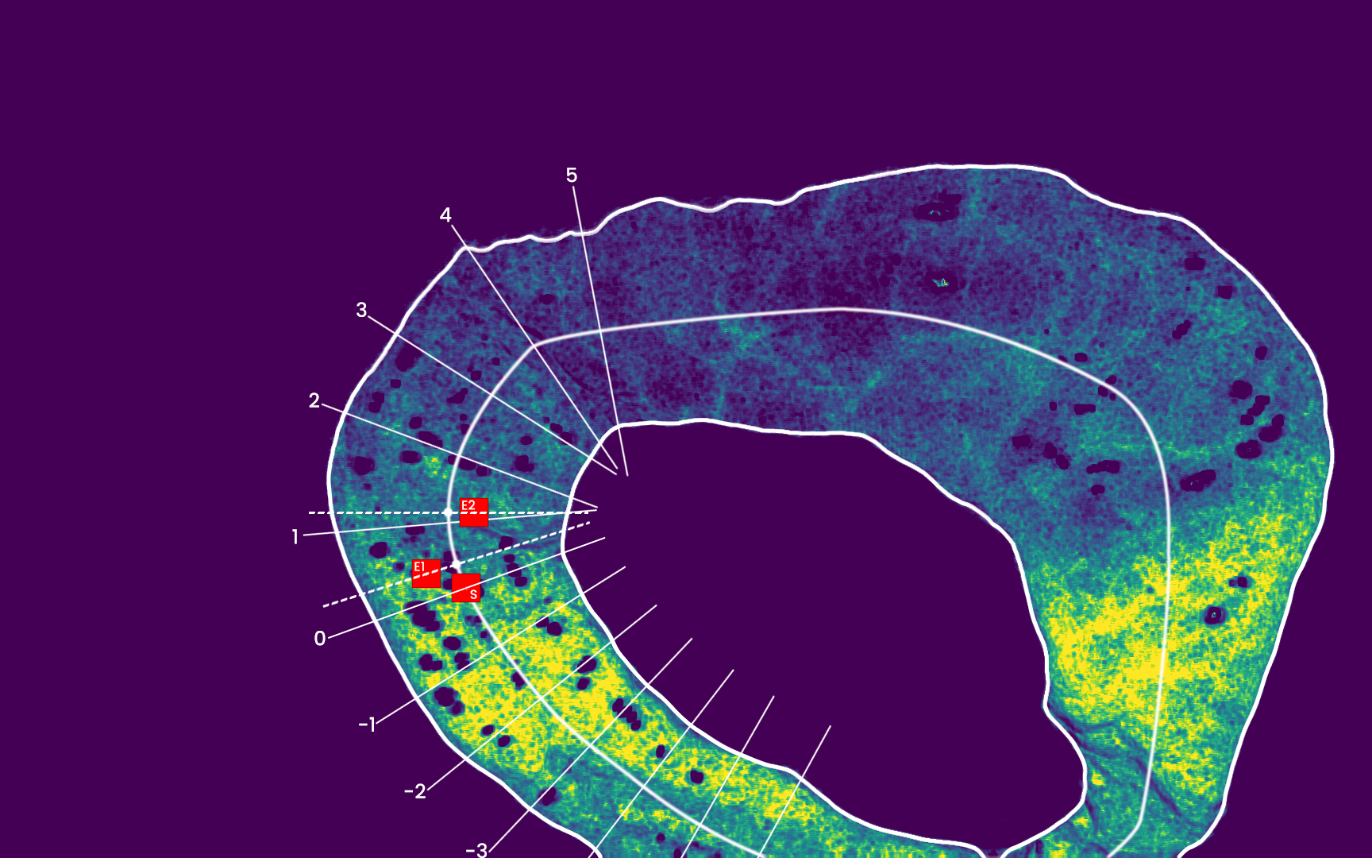


**Figure S2** | Determination of the midline on the intestinal loop was calculated by the distance between the outlines divided by two. Subsequently, perpendicular lines were positioned on the midline, intersecting the centre of the Regions of Interest (ROIs). The distance between ROIs was measured in pixels, considering the distance over the midline. A conversion factor of 85 pixels equals 1 cm was used, as measured from a reference image.


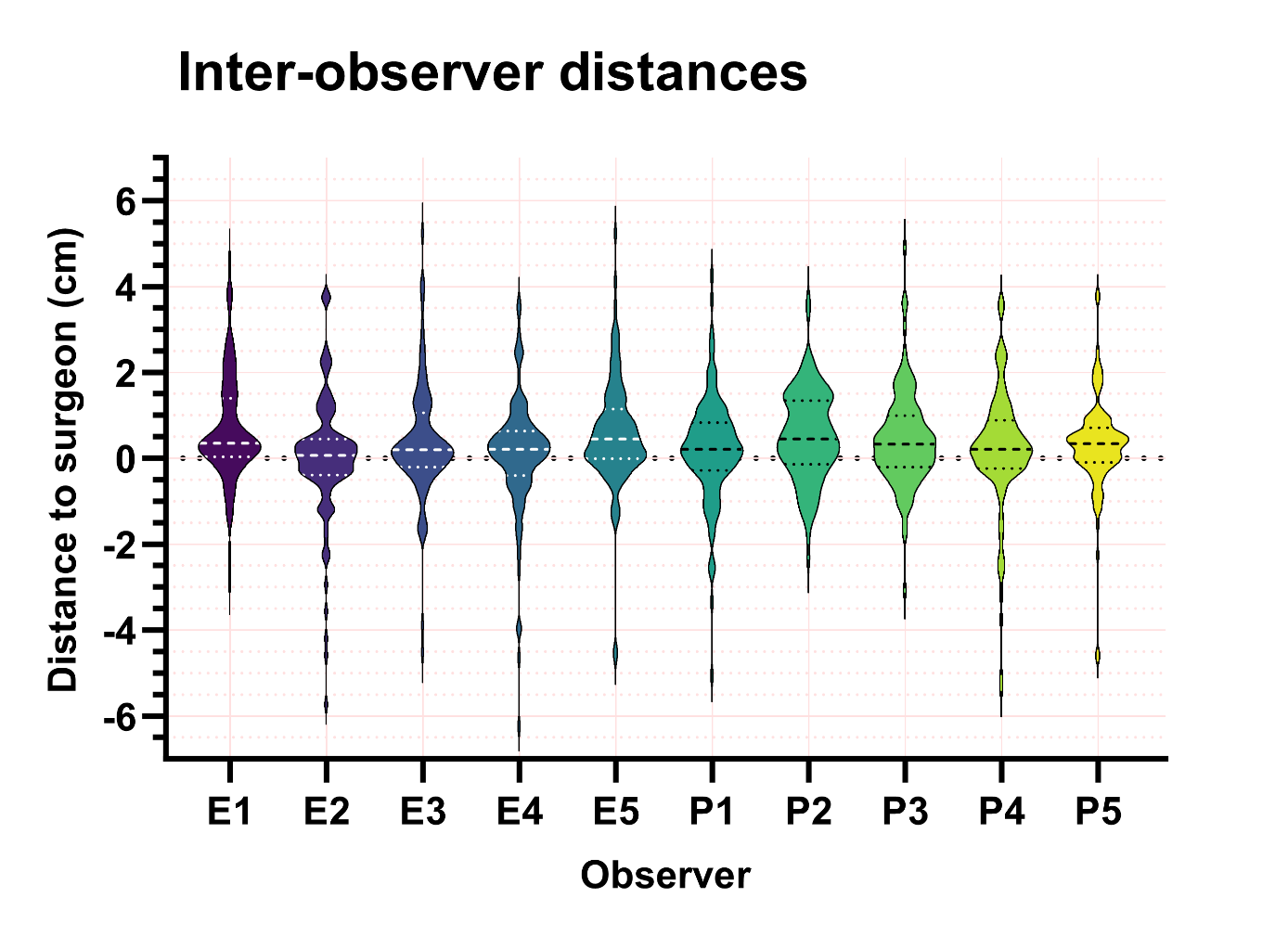
**Figure S3** | Violin plot of distances to the operating surgeon per observer. E_x_ = expert observer; P_x_ = Physician.


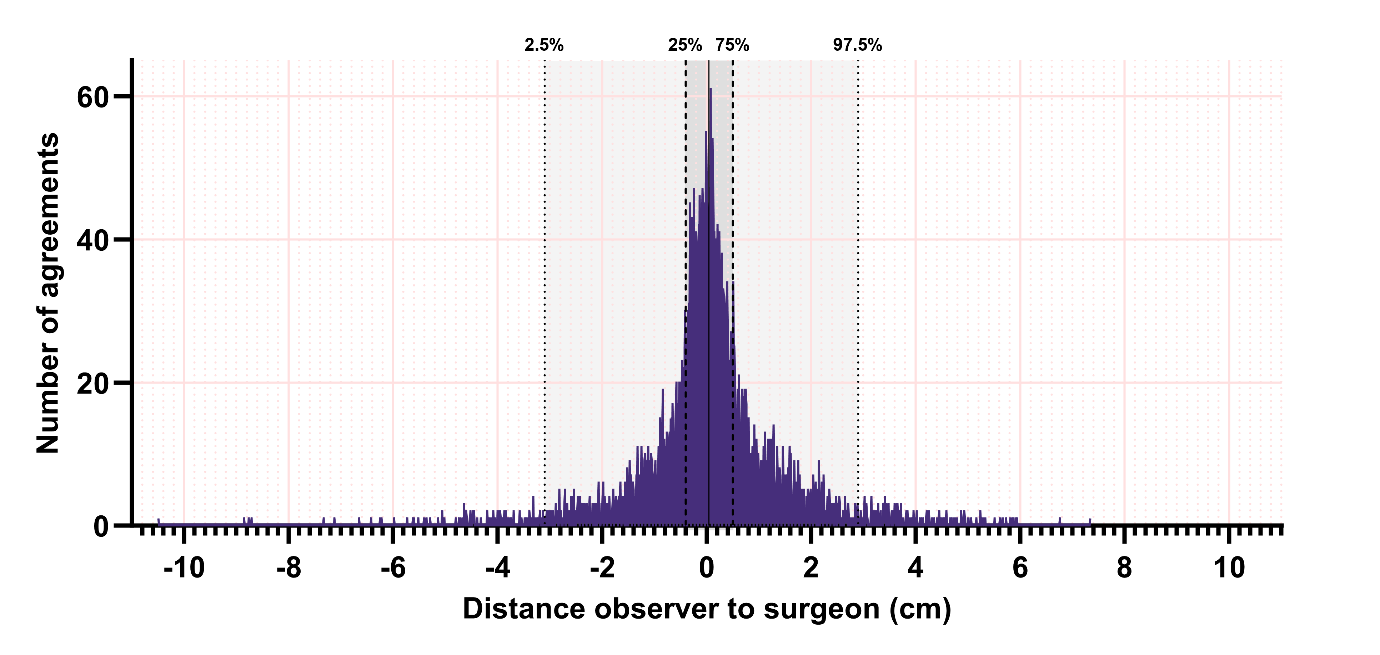


**Figure S4** | Histogram of the distribution of inter-observer distances. All experts and physicians were measured against the surgeon. 50% Of all measurements falls between -0.40 and 0.50 cm from the surgeon, with a mean of 0.04 cm. The 95% Confidence Interval lies between -3.10 and 2.9 cm.

## 2. Overview of P-values for LSPU and lactate levels.

**Table S1 |** Overview of all P-values between Regions of Interest (ROI) over time for both Laser Speckle Perfusion Units (LSPU) and lactate levels (mmol/L). The upper right half of the table represents P-values based on LSPU calculations, whereas the lower left half represents P-values based on lactate levels.Values below 0.05 area shown on a (light or dark) blue background.

**
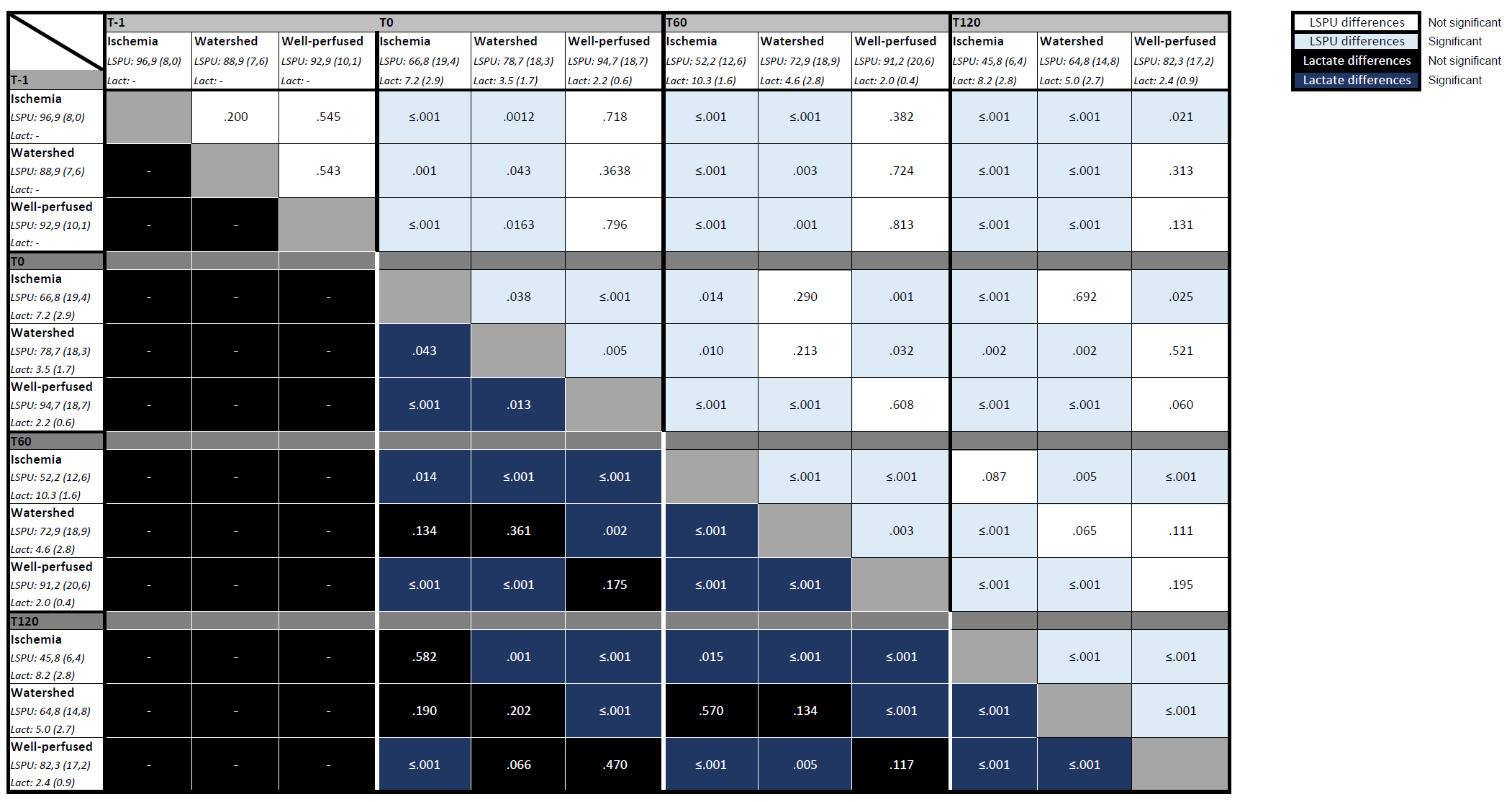
**
